# Citation Context Analysis of Autism Mortality and Suicide Findings From Hirvikoski’s Landmark Study

**DOI:** 10.1001/jamanetworkopen.2024.61953

**Published:** 2025-02-17

**Authors:** Brittany N. Hand, Melica Nikahd, Bethany J. Wolf, J. Madison Hyer, Anne Longo, Daniel Gilmore, Lauren Bishop

**Affiliations:** 1 Division of General Internal Medicine, The Ohio State University, Columbus; 2 Center for Biostatistics, The Ohio State University, Columbus; 3 Division of Biostatistics, Medical University of South Carolina, Charleston; 4 School of Health and Rehabilitation Sciences, The Ohio State University, Columbus; 5 Sandra Rosenbaum School of Social Work, University of Wisconsin–Madison; 6 Waisman Center, University of Wisconsin–Madison

## Abstract

This systematic review evaluates the accuracy of citations to a landmark study on premature mortality among autistic people.

## Introduction

Understanding health outcomes, including mortality, is a high priority for the autistic community.^1^ High-profile publications and government reports state “people with autism die 16 years earlier”^2^ or have a “54-year life expectancy,”^3^ citing Hirvikoski and colleagues’^4^ landmark study of mortality among autistic people. Life expectancy is estimated using life table models or by calculating average age of death after 100% of a population has died; yet, less than 3% of autistic people died during Hirvikoski and colleagues’^4^ observation period. Notably, underdiagnosis of autism among adults today reduces the generalizability of existing mortality estimates to future generations of autistic people. Age-related cohort effects in Hirvikoski and colleagues’^4^ sample also likely underestimate deaths from chronic conditions such as cardiovascular disease. Thus, drawing conclusions about life expectancy or leading causes of death for all autistic people misrepresents Hirvikoski and colleagues’^4^ findings. Indeed, more recent rigorous studies of life expectancy^5^ among autistic people found less extreme age of death disparities between autistic and nonautistic decedents. To systematically determine the degree to which the mortality findings in Hirvikoski et al^4^ were represented accurately by citing authors, we conducted a citation context analysis.^6^

## Methods

For this systematic review, we searched 6 databases to identify articles that cited Hirvikoski et al.^4^ We included full-text, peer-reviewed articles in English. Two team members (D.G. and A.L.) extracted data for each article (eTable 1 in Supplement 1), including all passages citing Hirvikoski et al.^4^ Then, we categorized articles as correct or accurate, incorrect or misleading, or irrelevant representations of the findings from Hirvikoski and colleagues^4^ (eTable 2 in Supplement 1). Descriptive statistics summarized included articles, and χ^2^ tests assessed associations between specific findings cited and article categorization. Cohen κ measured interrater reliability (κ = 0.73). The eMethods in Supplement 1 include additional details. We followed the PRISMA reporting guideline.

## Results

We included 506 articles (Table 1; eFigure in Supplement 1). Most articles (356 [70.4%]) were incorrect or misleading, 127 (25.1%) were correct or accurate, and 23 (4.5%) were irrelevant. Most authors (278 [54.9%]) cited all-cause mortality findings, 214 (42.3%) cited suicide findings, and 57 (11.3%) cited other cause-specific mortality findings. Articles citing all-cause mortality findings were significantly more likely to be categorized as incorrect or misleading, while those citing suicide findings were significantly more likely to be categorized as correct or accurate (Table 1). Citing other cause-specific mortality findings was not significantly associated with article categorization as correct or accurate or as incorrect or misleading.

**Table 1.** Characteristics of Included Articles

Characteristic	Studies, No. (%)			
	Correct or accurate (n = 127)	Incorrect or misleading (n = 356)	Not relevant (n = 23)	Total (N = 506)
Article primary topic				
Suicide	32 (25.2)	46 (12.9)	0	78 (15.4)
Mortality	9 (7.1)	14 (3.9)	1 (4.3)	24 (4.7)
Other	86 (67.7)	296 (83.1)	22 (95.7)	404 (79.8)
Autism or IDD journal				
Yes	55 (43.3)	156 (43.8)	5 (21.7)	216 (42.7)
No	72 (56.7)	200 (56.2)	18 (78.3)	290 (57.3)
Article type				
Review^a^	13 (10.2)	29 (8.1)	2 (8.7)	44 (8.7)
Narrative review	9 (7.1)	28 (7.9)	0	37 (7.3)
Original research^b^	81 (63.8)	229 (64.3)	19 (82.6)	329 (65.0)
Letter to the editor	4 (3.1)	7 (2.0)	0	11 (2.2)
Editorial	1 (0.8)	18 (5.1)	1 (4.3)	20 (4.0)
Commentary	9 (7.1)	25 (7)	0	34 (6.7)
Study protocol	3 (2.4)	7 (2)	1 (4.3)	11 (2.2)
Other	7 (5.5)	13 (3.7)	0	20 (4.0)
Hirvikoski findings cited^c^				
All-cause mortality	49 (38.6)^d^	229 (64.3)^d^	0	278 (54.9)
Suicide	72 (56.7)^d^	142 (39.9)^d^	0	214 (42.3)
Other cause-specific mortality	12 (9.4)^e^	45 (12.6)^e^	0	57 (11.3)
Citations, median (IQR)	13 (4-38)	15 (3-50)	11 (7-34)	14 (4-48)
Altmetric Score, median (IQR)	15 (4-44)	15 (3-51)	6 (1-13)	14 (3-47)
Impact factor, median (IQR)	4.7 (2.9-5.7)	4.7 (3.7-5.9)	2.8 (2.6-6.2)	4.7 (3.4-5.9)

Abbreviation: IDD, intellectual or developmental disability.

^a^ Includes systematic reviews, scoping reviews, and meta-analyses.

^b^ Includes original research articles, short reports of original research, research letters, and conference proceedings papers.

^c^ Categories are not mutually exclusive.

^d^ *P* < .001.

^e^ *P* = .44.

Among 127 articles with correct or accurate representations, 71 (55.9%) cited the higher suicide mortality rate, 48 (37.8%) cited the higher all-cause mortality rate, and 10 (7.9%) broadly cited health inequities experienced by autistic people (Table 2). Among 356 articles with incorrect or misleading representations, the most common incorrect representation was using the term premature mortality (191 [53.7%]). Other incorrect representations included conflating suicidality with death by suicide (61 [17.1%]), making unsupported conclusions about autistic peoples’ life expectancy (59 [16.6%]), and overgeneralizing findings on decedent mean age of death (56 [15.7%]). Some articles misrepresented odds ratios comparing suicide deaths between autistic and nonautistic people, either by conflating the largest odds ratio with the leading cause of death (30 [8.4%]) or by inaccurately or incompletely reporting the odds ratio (33 [9.3%]).

**Table 2.** Representation of Key Findings From Hirvikoski et al by Citing Authors

Category	Brief rationale^a^	Representative composite example^b^	Articles, No. (%)	Median (IQR)	
				Citations^c^	Altmetric Score
**Correct or accurate^d^**					
Higher suicide rate	Autistic people had 7.55 times greater odds of death by suicide than the control group.	Autistic adults experience higher suicide risk compared with nonautistic adults.	71 (55.9)	16 (4-49)	17 (3-60)
Higher mortality rate	Autistic people had 2.56 times greater odds of all-cause mortality than the control group.	Mortality rates for autistic adults are higher than those for nonautistic adults.	48 (37.8)	13 (3-30)	12 (4-34)
Health inequities	The finding that autistic people had 2.56 times greater odds of death underscores health disparities or inequities.	Autistic people have poorer health outcomes than nonautistic peers.	10 (7.9)	8 (2-20)	12 (3-55)
Other correct or accurate	Representation correct but not represented previously.	Autistic people are twice as likely to die from cancer as nonautistic people.	7 (5.5)	6 (3-35)	9 (5-16)
**Incorrect or misleading^d^**					
Premature mortality	Premature mortality is the proportion of deaths occurring prior to the national average age of death.	Autistic adults are at greater risk of premature mortality than the general population.	191 (53.7)	16 (4-53)	16 (3-49)
Suicidality vs suicide	The study examined death by suicide, which is distinct from suicidality.	Suicidality is common among autistic individuals.	61 (17.1)	25 (4-62)	21 (6-64)
Life expectancy	Life expectancy is the age by which about 50% of a population have died, but only 2.6% of autistic people died during the study.	Autistic people have shorter life spans than the general population.	59 (16.6)	14 (3-40)	19 (2-55)
Overgeneralized mean age of death	Mean age of death is only reflective of the minority of autistic participants who died during the study.	Autistic people die 16 y earlier than the general population.	56 (15.7)	20 (3-55)	17 (3-93)
Prevalence of health conditions	Co-occurring health conditions were not quantified, only causes of death.	Autistic people have higher risk of physical health conditions.	38 (10.7)	11 (3-57)	16 (4-68)
Odds of suicide inaccurate	These representations reflect rounding errors or omit key details needed for accurate representation.	Autistic adults are 9 times more likely to die by suicide than the general population.	33 (9.3)	14 (4-50)	18 (4-49)
Suicide leading cause of death	Suicide was the cause of death for which the odds ratio was largest, but not the cause of the largest proportion of deaths.	Suicide is the leading cause of death in autistic people.	30 (8.4)	43 (11-99)	19 (6-64)
Causal language	Inferences about causality cannot be made from an observational case-control cohort study.	Autism increases the risk of suicide mortality.	20 (5.6)	17 (3-102)	11 (3-73)
Other incorrect or misleading	Representation of findings incorrect or misleading but not represented previously.	Loneliness and isolation predict suicide in autistic people.	15 (4.2)	10 (3-53)	10 (2-31)
**Irrelevant**					
Irrelevant	Citations not clearly relevant to the findings of Hirvikoski et al.^4^	Autism is a developmental health condition.	23 (4.5)	11 (7-34)	6 (1-13)

^a^ See the eMethods and eTable 2 in Supplement 1 for full rationale.

^b^ Representative composite examples were used to characterize each subcategory without putting undue emphasis on specific articles and authors.

^c^ Citations are the number of times articles included in this study have been cited by subsequent authors.

^d^ Within the categories of correct or accurate and incorrect or misleading, articles may be represented in more than 1 subcategory (eg, an article may correctly cite both the higher suicide rate and higher mortality rate findings from Hirvikoski et al^4^).

## Discussion

Widespread misrepresentation of findings from Hirvikoski et al,^4^ particularly the erroneous portrayal of 16-year reduced life expectancy, underscores the critical importance of research dissemination accuracy and provides an empirical example of scientific communication and miscommunication beyond the intent of the original authors. This misrepresentation has been amplified by high-impact publications that conflate the study’s findings on decedent mean age of death with unsupported claims of premature mortality and reduced life expectancy. While not the fault of the original authors, the inaccurate portrayal of findings by citing authors has potential repercussions for autistic people, including psychological distress, altered retirement planning, insurance denials, and exacerbated health disparities. We assert there is a moral imperative for authors, peer reviewers, and journals to accurately represent findings from cited studies to avoid perpetuating misinformation and associated harms.

## Notes

Supplement 1.
eMethods.
**eFigure.** Flow Chart of Article Selection **eTable 1**. Article Type Classification **eTable 2.** Correct/Accurate or Incorrect/Misleading Representations of Key Findings From Hirvikoski et al

Supplement 2.
Data Sharing Statement
