# Supplementary material for: Citation Context Analysis of Autism Mortality and Suicide Findings From Hirvikoski’s Landmark Study
Source: JAMA Netw Open. 2025 Feb 17;8(2):e2461953. doi: 10.1001/jamanetworkopen.2024.61953 (PMC11833514; doi:10.1001/jamanetworkopen.2024.61953)
Supplement: Supplement 1. — eMethods. eFigure. Flow Chart of Article Selection eTable 1. Article Type Classification eTable 2. Correct/Accurate or Incorrect/Misleading Representations of Key Findings From Hirvikoski et al [file jamanetwopen-e2461953-s001.pdf]

# Supplemental Online Content

Hand BN, Nikahd M, Wolf B, et al. Citation context analysis of autism mortality and suicide findings from Hirvikoski's landmark study. *JAMA Netw Open.* 2025;8(2):e2461953. doi:10.1001/jamanetworkopen.2024.61953

**eMethods.**

**eFigure.** Flow Chart of Article Selection

**eTable 1** . Article Type Classification

**eTable 2.** Correct/Accurate or Incorrect/Misleading Representations of Key Findings From Hirvikoski et al

This supplemental material has been provided by the authors to give readers additional information about their work.

© 2025 Hand BN et al. *JAMA Network Open*.

**eMethods.**

Search Strategy

We forward searched six research databases (MEDLINE, Scopus, CINAHL, PsychINFO, Web of Science, and PubMed) to identify articles that cited Hirvikoski et al., 2016. We imported the articles into Covidence and removed duplicates. The first author conducted the searches and identified articles published during 2015 (as Hirvikoski et al., 2016 was available ahead of print) through January 31, 2024.

Article Selection

Included articles must have: (1) had a full text available (i.e., not just a published abstract); (2) been published in a peer-reviewed journal; and (3) been published in English. We excluded articles if they were: (1) published abstracts only; (2) not published in peer-reviewed journals (e.g., dissertations, websites, books); or (3) not in English. Two independent study team members reviewed each article for inclusion or exclusion, with disagreements resolved by the first author. eFigure 1 details the article selection process.

Data Extraction

Two team members independently extracted data for each article and BH made final determinations. Data extracted from each article included the first author's last name, publication year, paper title, Altmetric score, number of citations according to Google Scholar, and all passages of text where Hirvikoski et al., 2016 was cited. We also extracted whether the passages of text citing Hirvikoski et al., 2016 referenced findings pertaining to all-cause mortality, death by suicide, and/or other specific causes of death as well as whether the primary topic of the article was suicide-related (including topics like suicidality, suicidal ideation, suicide attempts, suicide, self-harm, suicidal behaviors, etc.), mortality-related (including topics like mortality, life-expectancy, death, etc.), or "other." Last, we classified the article type as: (1) original research, (2) systematic reviews, (3) narrative reviews, (4) commentaries, (5) editorials, (6) letters to the editor, (7) study protocols, and (8) other articles (eTable 1).

We also extracted information about the publishing journal, including the journal name, whether the journal has a primary emphasis on autism and/or intellectual and developmental disabilities, and the impact factor as of the year the article was published according to Scimago Journal & Country Rank.

© 2025 Hand BN et al. *JAMA Network Open*.

## Article Evaluation

BH and LB collaboratively reviewed a random 20% sample of articles and developed an initial codebook to guide article evaluation. The initial codebook included examples of correct/accurate interpretations of Hirvikoski et al., 2016's findings, incorrect/misleading interpretations, irrelevant citations (i.e., citation is in a sentence that is not clearly related to the findings of Hirvikoski et al., 2016) and an "unsure" category. We consulted with BW, an expert biostatistician in mortality risk estimation, to clarify the examples in the "unsure" category and finalize the codebook (eTable 2). BH and LB independently coded all included articles using the codebook and resolved disagreements via discussion. In instances where Hirvikoski et al., 2016 was cited more than once in an article, if at least one instance was incorrect/misleading, the article was categorized as incorrect/misleading. Articles categorized as "incorrect/misleading" may have also contained correct/accurate representations, but these were not coded.

## Ethical Review of Study

This research is exempt from Institutional Review Board review due to the use of publicly available published research articles.

## Protocol and Registration

As we are unaware of established reporting guidelines for citation context analyses, we followed the PRISMA 2020 checklist for systematic reviews as it was the closest match. The protocol for this citation context analysis was not registered.

## Limitations

Findings should be interpreted considering several methodological factors. Our study focused specifically on articles published in English, in peer-reviewed journals. Examinations of how the findings of Hirvikoski et al., 2016 are represented in news media, social media, gray literature, books, or in languages other than English, was beyond the scope of this study but may have a considerable impact on public perception. Social media posts shared on YouTube, Instagram, and private Twitter or Facebook pages, would not be captured in Altmetric score data, potentially underestimating article reach. Although our categorization

© 2025 Hand BN et al. *JAMA Network Open*.

procedures were conducted by multiple reviewers, they carry the possibility of bias. To minimize risk of bias, in instances where either BH or LB was a co-author on an included article, the other categorized the article as correct/accurate or incorrect/misleading. In instances where both BH and LB were co-authors on the article, BW provided the categorization. Examining longitudinal patterns in the representation of key findings of Hirvikoski et al., 2016 was beyond the scope of the present study, but will be important for future work. Of note, this study focused on articles citing Hirvikoski et al., 2016; future studies examining how representations of findings from other mortality studies are warranted to provide a more holistic picture of how mortality findings are discussed in the literature. Despite these limitations, this study constitutes a comprehensive citation context analysis of the most cited, largest study of mortality among autistic people since its publication in 2016.

© 2025 Hand BN et al. *JAMA Network Open*.

**eFigure.** Flow Chart of Article Selection

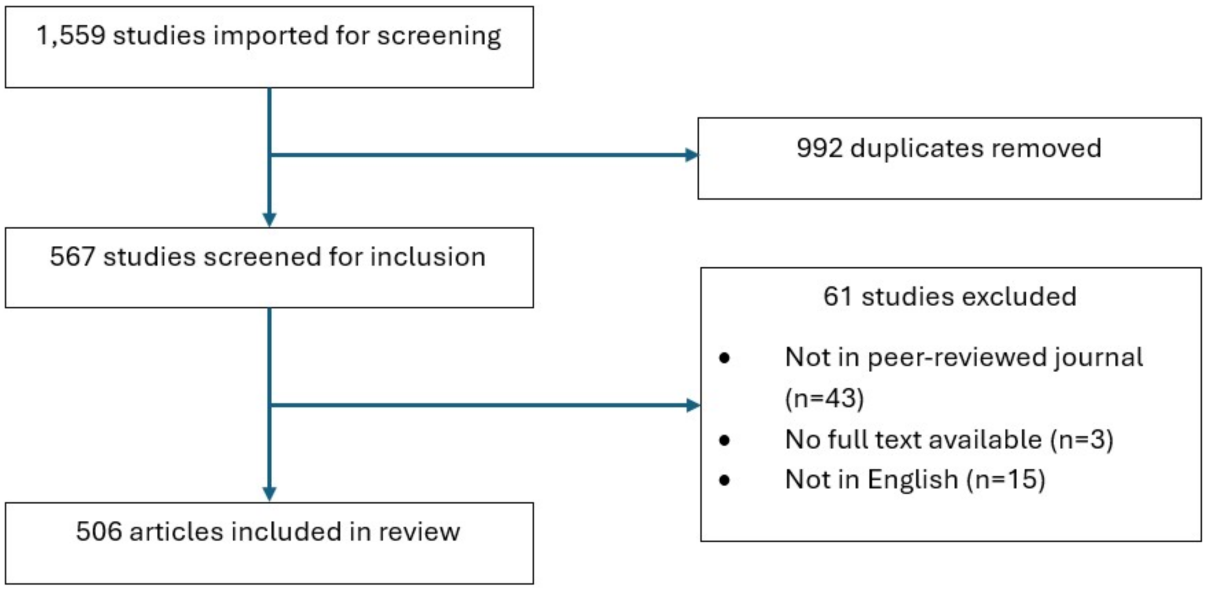


© 2025 Hand BN et al. *JAMA Network Open*.

**eTable 1**. Article Type Classification

| Article type | Description |
|---|---|
| Original research | Reports original research methods and results, including short-reports, full-length articles, research letters, and conference proceedings papers |
| Systematic review | Summarizes the literature in a methodologically formal way such as a systematic review, meta-analysis, or scoping review |
| Narrative review | Review or summarize the literature without structured formal methods |
| Commentary | Free-standing articles pointing out a problem, expressing views or opinions, and provoking scholarly dialogue (i.e., articles classified as commentaries, perspectives, viewpoints, opinion pieces) |
| Editorial | Remark on an article or group of articles published in the journal and are typically authored by the journal editor(s) |
| Letter to the editor | Provide feedback from readers about an article regarding whether they have different interpretations of the data or which to provide constructive comments. |
| Study protocol | Describe the guidelines and procedures for conducting an original research project or systematic review |
| Other | Articles that do not readily fit into one of the above categories, including but are not limited to, continuing medical education articles, clinical practice guidelines, workshop/working group reports, roundtable discussions, etc. |

© 2025 Hand BN et al. *JAMA Network Open*.

**eTable 2.** Correct/Accurate or Incorrect/Misleading Representations of Key Findings From Hirvikoski et al

| Category | Description | Full Rationale |
|---|---|---|
| **Correct/Accurate** | | |
| Higher suicide rate | (increased OR higher OR elevated OR greater) AND (risk of OR rate of OR likelihood of OR odds of) AND (suicide OR death by suicide OR death by suicide OR suicide completion) | Autistic people had 7.55 times greater odds of death by suicide than the matched control group. Death by suicide was more common among autistic people without an intellectual disability (relative to those with ID). Rates of suicide were more uniform between males and females in the autistic sample (0.32% females vs 0.30% males), leading to a higher relative risk in autistic females compared to matched control females. |
| Higher mortality rate | (increased OR higher OR elevated OR greater) AND (mortality OR mortality risk OR mortality rates OR death) | Autistic people had 2.56 times greater odds of all-cause mortality than the matched control group. |
| Health inequities | Health inequities OR health disparities | The finding that autistic people had 2.56 times greater odds of death underscores health disparities/inequities. |
| Other correct/ accurate | Correct representation of findings that does not readily fit into one of the above categories | Some representations of findings were correct but did not occur frequently enough to constitute a stand-alone category. |
| **Incorrect/Misleading** | | |
| Premature mortality | (early OR premature) AND (mortality OR death) | Premature mortality is the proportion of deaths occurring prior to the national average age of death, which was not quantified. Without doing a time to event analysis and given the limited proportion of people who died, there is insufficient evidence to support this. |
| Suicidality vs. suicide | Suicidality OR suicidal behaviors OR self-injurious behaviors OR suicidal ideation | Hirvikoski et al., 2016 examined the outcome death by suicide, which is meaningfully different from these terms. |
| Life expectancy | Life expectancy OR life span | Life expectancy is the age by which about 50% of a population have died. Given that only 2.6% of autistic people died during the study, generalizations about life expectancy about the entire autistic sample or autistic people more broadly cannot be drawn. |

© 2025 Hand BN et al. *JAMA Network Open*.

| | | |
|---|---|---|
| Overgeneralized mean age of death | References (mean age of death OR differences in mean age of death between autistic and non-autistic people) WITHOUT clarifying minority of sample died during study | Mean age of death is only reflective of the minority of autistic participants who died during the study (n=706) and does not generalize to the entire autistic sample (n=27,122). |
| Prevalence of health conditions | Autistic people experience more mental and physical health conditions than non-autistic peers OR Co-occurring health conditions explain excess mortality among autistic people | Co-occurring health conditions were not examined in Hirvikoski et al., 2016. Only causes of death were examined. |
| Odds of suicide inaccurately reported | Autistic people are (9 times more likely to die by suicide than non-autistic people WITHOUT clarifying this estimate is only for autistic people without intellectual disability OR 7 times more likely to die by suicide than non-autistic people) OR Autistic females (without ID are at the greatest risk of death by suicide OR are more likely to die by suicide than autistic males) | The autistic group (as a whole) had 7.55 times greater odds of death by suicide than controls. Autistic people without ID had 9.4 times greater odds of death by suicide relative to matched controls, while those with intellectual had 2.41 times greater odds of death by suicide than matched controls. The study did not stratify by both sex and ID status for cause-specific mortality rates. There were similar rates of suicide between autistic females (0.32%) and autistic males (0.30%), indicating the absence of sex-based differences observed in the general population; as a result, autistic females had a higher relative risk of suicide compared to non-autistic females. |
| Suicide is a leading cause of death | Suicide is the (leading OR most common OR second leading) cause of (death OR premature death) among autistic people | Suicide was the cause of death for which the odds ratio was largest, but was not the cause of the largest proportion of deaths. |
| Causal language | Uses causal language | Inferences about causality cannot be made from an observational case control cohort study. |
| Other incorrect/ misleading | Incorrect or misleading representation of findings that does not readily fit into one of the above categories | Some representations of findings were incorrect or misleading, but did not occur frequently enough to constitute a stand-alone category. |
| **Irrelevant** | | |
| Irrelevant | Not clearly relevant to the findings of Hirvikoski et al., 2016 | Citations not clearly relevant to the findings of Hirvikoski et al., 2016 cannot be classified as correct or incorrect representations. |

ID = Intellectual disability

© 2025 Hand BN et al. *JAMA Network Open*.
