# Supplementary material for: Citation Context Analysis of Autism Mortality and Suicide Findings From Hirvikoski’s Landmark Study
Source: JAMA Netw Open. 2025 Feb 17;8(2):e2461953. doi: 10.1001/jamanetworkopen.2024.61953 (PMC11833514; doi:10.1001/jamanetworkopen.2024.61953)
Supplement: Supplement 2. — Data Sharing Statement [file jamanetwopen-e2461953-s002.pdf]

Data Sharing Statement

Hand. Citation Context Analysis of Autism Mortality and Suicide Findings From Hirvikoski's Landmark Study. *JAMA Netw Open*. Published February 17, 2025. doi:10.1001/jamanetworkopen.2024.61953

Data
Data available: Yes
Data types: Data (not involving human participants), Data dictionary, Other (please specify)
Additional Information: Codebook
How to access data: De-identified data (i.e., absent of author names, article titles, journal names, and direct quotes) is publicly available through our institutional data repository (https://kb.osu.edu/handle/1811/105470). An identifiable version of the dataset is available via request to the corresponding author at brittany.hand@osumc.edu, pending IRB approval/exemption and completion of a data sharing agreement.
When available: With publication

Supporting Documents
Document types: None

Additional Information
Who can access the data: Researchers with IRB approval/exemption
Types of analyses: for any purpose
Mechanisms of data availability: with a signed data access agreement
